# A pilot cross-sectional investigation of symptom clusters and associations with patient-reported outcomes in Myalgic Encephalomyelitis/Chronic Fatigue Syndrome and Post COVID-19 Condition

**DOI:** 10.1007/s11136-024-03794-x

**Published:** 2024-10-03

**Authors:** Breanna Weigel, Natalie Eaton-Fitch, Kiran Thapaliya, Sonya Marshall-Gradisnik

**Affiliations:** 1 National Centre for Neuroimmunology and Emerging Diseases, Griffith University, 1 Parklands Drive, Southport Gold Coast, Brisbane, QLD 4222 Australia; 2 Consortium Health International for Myalgic Encephalomyelitis, Griffith University, Gold Coast, Brisbane, QLD 4222 Australia; 3 School of Pharmacy and Medical Sciences, Griffith University, Gold Coast, Brisbane, QLD 4222 Australia

**Keywords:** Myalgic Encephalomyelitis/Chronic Fatigue Syndrome, Post COVID-19 Condition, Post-Acute Sequelae of COVID-19, Long COVID, Quality of life

## Abstract

**Background:**

Myalgic Encephalomyelitis/Chronic Fatigue Syndrome (ME/CFS) is associated with long-term disability and poor quality of life (QoL). Cardinal ME/CFS symptoms (including post-exertional malaise, cognitive dysfunction and sleep disturbances) have been observed in Post COVID-19 Condition (PCC). To gain further insight into the potential role of ME/CFS as a post-COVID-19 sequela, this study investigates associations between symptoms and patient-reported outcomes, as well as symptom clusters.

**Methods:**

Participants included Australian residents aged between 18 and 65 years formally diagnosed with ME/CFS fulfilling the Canadian or International Consensus Criteria or PCC meeting the World Health Organization case definition. Validated, self-administered questionnaires collected participants’ sociodemographic and illness characteristics, symptoms, QoL and functional capacity. Associations between symptoms and patient-reported outcomes were investigated with multivariate linear regression models. Hierarchical cluster analysis was performed to identify symptom clusters.

**Results:**

Most people with ME/CFS (pwME/CFS) and people with PCC (pwPCC) were female (*n* = 48/60, 80.0% and *n* = 19/30, 63.3%, respectively; *p* = 0.12). PwME/CFS were significantly younger (*x̄*=41.75, *s* = 12.91 years) than pwPCC (*x̄*=48.13, *s* =10.05 years; *p* =0.017). Autonomic symptoms (notably dyspnoea) were associated with poorer scores in most patient-reported outcome domains for both cohorts. None of the four symptom clusters identified were unique to ME/CFS or PCC. Clusters were largely delineated by the presence of gastrointestinal and neurosensory symptoms, illness duration, ME/CFS criteria met and total symptoms.

**Conclusions:**

Illness duration may explain differences in symptom burden between pwME/CFS and pwPCC. PCC diagnostic criteria must be refined to distinguish pwPCC at risk of long-term ME/CFS-like illness and subsequently deliver necessary care and support.

**Supplementary Information:**

The online version contains supplementary material available at 10.1007/s11136-024-03794-x.

## Abstract (plain language summary)

Post COVID-19 Condition (PCC), or Long COVID, refers to the ongoing symptoms experienced after acute COVID-19 illness. The symptoms reported by people with PCC (pwPCC) resemble Myalgic Encephalomyelitis/Chronic Fatigue Syndrome (ME/CFS). However, existing diagnostic criteria for PCC are broad and cannot discern PCC subtypes (such as pwPCC experiencing ME/CFS-like illness), which may require different approaches to care. This study contributes to improving PCC case criteria and approaches to care for both ME/CFS and PCC by investigating the relationships between symptoms and patient-reported outcomes among pwPCC and people with ME/CFS (pwME/CFS).

For both cohorts, symptoms had a negative relationship with all aspects of health. Autonomic symptoms, notably breathing issues, returned the most negative associations. Pain, flu-like symptoms and lack of temperature control appeared more burdensome among pwPCC. These symptoms may signify the early stages of ME/CFS. Symptom clusters were also identified for the first time among a combined cohort of pwME/CFS and pwPCC in this study. Importantly, none of the symptom clusters were specific to ME/CFS or PCC. Instead, symptom clusters were defined by the prevalence of gastrointestinal and neurosensory symptoms, illness duration, ME/CFS criteria met and the total number of symptoms.

This study suggests that the few differences between ME/CFS and PCC may be explained by illness duration. These findings further implicate ME/CFS as a potential post-COVID-19 outcome. As ME/CFS is associated with profound reductions in quality of life and functioning, identifying pwPCC experiencing ME/CFS-like illness through refined diagnostic criteria must be prioritised to ensure the delivery of necessary care.

**Supplementary Information:**

The online version contains supplementary material available at 10.1007/s11136-024-03794-x.

## Introduction

Myalgic Encephalomyelitis/Chronic Fatigue Syndrome (ME/CFS) is a chronic multi-systemic illness associated with substantial reductions in quality of life (QoL) and profound functional impairments [1–6]. People with ME/CFS (pwME/CFS) experience poorer mental and emotional wellbeing, as well as a profound burden on physical health when compared with healthy people and the general population [2, 3, 7, 8]. The symptoms of ME/CFS are debilitating and impose considerable restrictions on one’s ability to participate in the activities of typical daily life [1, 4, 5, 9]. Post-exertional malaise – the worsening of symptoms following physical, mental or emotional exertion – is the cardinal symptom of ME/CFS [4–6, 9]. Other key symptoms include cognitive dysfunction, unrefreshed sleep, bodily pain and autonomic disturbances, such as thermostatic dysregulation, gastrointestinal upset and cardiorespiratory symptoms [4–6, 9].

The global prevalence of ME/CFS is approximately 1% [4–6, 10]. As investigations into an illness-specific biomarker for ME/CFS remain ongoing, diagnosis continues to rely on the fulfillment of case definitions [4, 9]. Currently, the preferred case definitions for diagnosing ME/CFS include the Canadian Consensus Criteria (CCC) [6] and International Consensus Criteria (ICC) [5]. There is no curative therapy for ME/CFS and recovery is reported in fewer than 10% of cases [4, 9, 11, 12]. The aetiopathogenesis of ME/CFS also remains elusive [4–6, 9]. Many environmental stressors, such as exposure to chemicals, trauma, stress and injury, have been identified as risk factors [11–13]. However, between 60% and 80% of pwME/CFS report a post-infectious illness onset [4, 14, 15].

Chronic multi-systemic illness reminiscent of ME/CFS has been observed among people with a history of Severe Acute Respiratory Syndrome Coronavirus-2 (SARS-CoV-2) infection [16–18]. Termed “Post COVID-19 Condition (PCC)”, this post-infectious sequela of acute COVID-19 illness consists of persistent symptoms for a minimum of 12 weeks according to the World Health Organization (WHO) case definition [19]. Between 40% and 60% of people with PCC (pwPCC) fulfil ME/CFS case criteria [20–22]. Additionally, the reported prevalence of post-exertional malaise among pwPCC ranges from 50% to 100% [20, 22–26].

Like ME/CFS, PCC is associated with poorer self-perceptions of overall health status and impairments in physical, mental and emotional wellbeing [22, 27, 28]. Whilst shared impairments in cell function have been identified among pwME/CFS and pwPCC [29], incomplete understanding of ME/CFS and PCC pathophysiology impedes definitive conclusions regarding the relationship of these two illnesses as the same or related, yet different, entities [16, 17, 30]. Additionally, PCC case definitions are broad and are unable to differentiate between illness subtypes [30–32]. Identifying COVID-19 survivors at risk of long-term illness reminiscent of ME/CFS is integral to guide the appropriate provision of care services and ensure a personalised approach to illness management [31–33].

The present study therefore serves to inform developments in PCC case definitions by comparing the illness burdens experienced by pwME/CFS and pwPCC in detail. Regression and cluster analyses were pursued to determine whether PCC exhibits distinct illness characteristics when compared with ME/CFS that may aid in differentiating subtypes of post-COVID-19 sequelae. Hence, this research may provide further insight into the role of ME/CFS in the illness trajectory of PCC.

This study also contributes to informing care pathways for ME/CFS and PCC by identifying associations between specific symptoms with QoL and functional capacity domains. Consequently, this research may highlight priority areas to be considered in the clinical management of ME/CFS and PCC to mitigate further QoL reductions and functional impairments. Finally, this study is the first to document symptom clusters and associations with patient-reported outcomes among an Australian cohort of pwME/CFS and pwPCC. By exemplifying the burden of ME/CFS and PCC on consumers in the Australian context, this research serves to guide national healthcare policy reforms and necessitate improved access to multidisciplinary care and support services for Australians who live with these conditions.

## Methods

### Study setting and participants

**Fig. 1:**
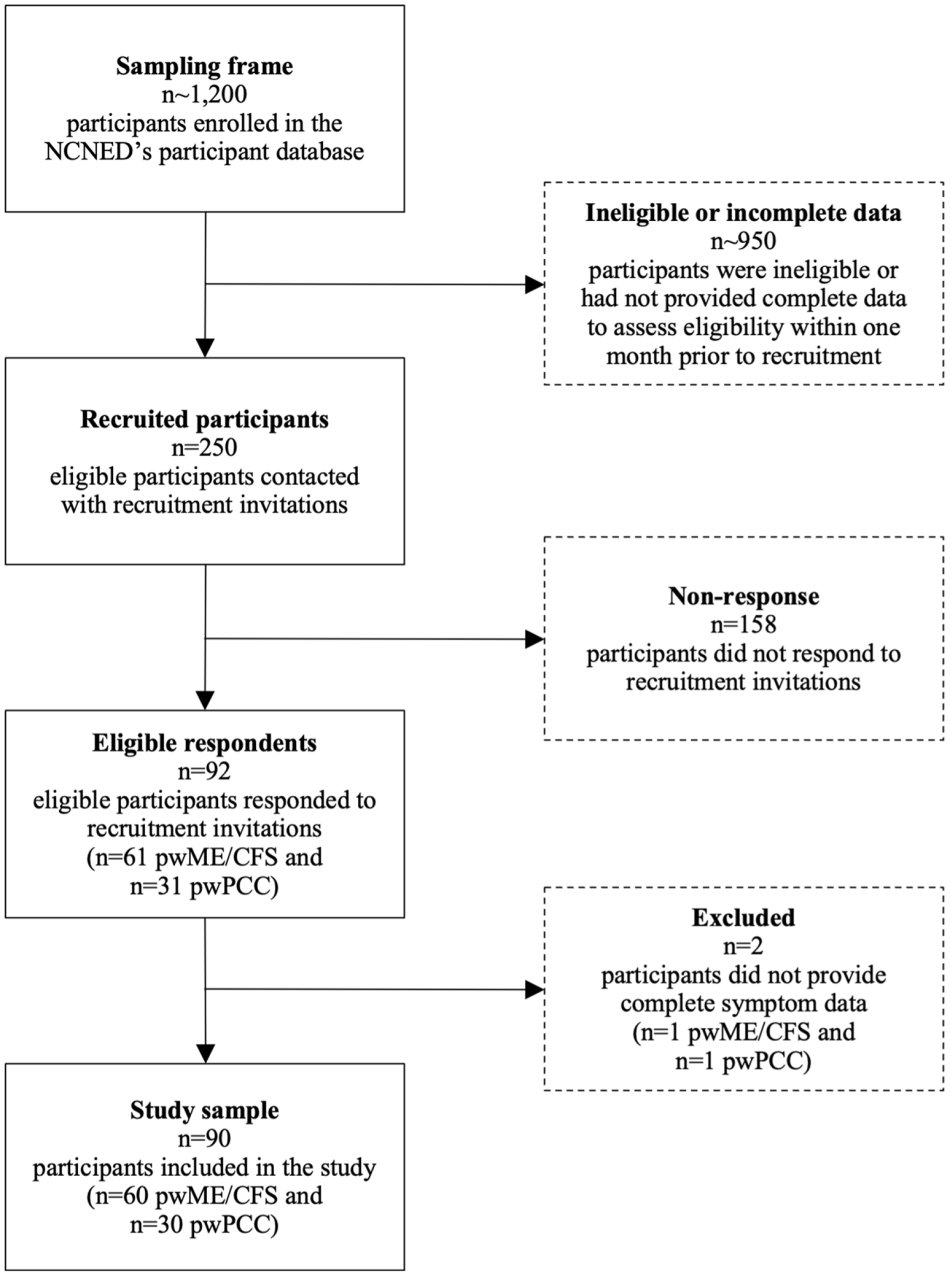
Participant recruitment and screening for eligibility. Figure generated with Microsoft Word. *Abbreviations NCNED* National Centre for Neuroimmunology and Emerging Diseases; *PwME/CFS* People/person with Myalgic Encephalomyelitis/Chronic Fatigue Syndrome; *PwPCC* People/person with Post COVID-19 Condition

Data was collected for this cross-sectional study between March 2021 and August 2022 from a cohort of pwME/CFS and pwPCC who participated in a previous research project at the National Centre for Neuroimmunology and Emerging Diseases (NCNED), Griffith University, Gold Coast, Queensland, Australia [22]. This dataset [22] was ascertained by screening the NCNED’s participant database, which comprised approximately 1,200 participants. Of the database participants, 250 were eligible and recruited. Among these participants, *n* = 61 pwME/CFS and *n* = 31 pwPCC participated in the research [22]. These participants were subsequently evaluated to determine their eligibility for further analysis in the present study (Fig. 1).

To be considered eligible, participants were required to be: (1) aged between 18 and 65 years; (2) a current Australian resident; and (3) formally diagnosed with ME/CFS or PCC by a physician. Additional eligibility criteria for pwME/CFS included: (a) a history of fulfilling at least one of the CCC [6] or ICC [5] within the last two years and (b) no history of acute COVID-19 illness prior to ME/CFS onset. Fulfilling the WHO case definition [19] was mandatory for pwPCC.

Participants who were currently smoking or pregnant were deemed ineligible. Additionally, participants were excluded if they reported a history of health concerns that may confound or explain their symptoms, QoL or functional capacity. Such exclusionary health concerns have been previously described [22] and include any formally diagnosed: (1) genetic, metabolic, immunological, neurological, cardiovascular or respiratory disease; (2) malignancy within the last five years; and (3) mental illness or other chronic multi-systemic or post-viral illness.

Comorbid dysautonomia or chronic pain conditions (such as Irritable Bowel Syndrome (IBS), Postural Orthostatic Tachycardia Syndrome and Fibromyalgia) were not considered exclusionary in the present study due to the notable overlap of the symptoms of these conditions with ME/CFS and post-viral illness [5, 6, 15, 34, 35]. Similarly, a concurrent or subsequent diagnosis of anxiety or depression was not considered exclusionary due to the frequent co-occurrence of secondary anxiety and depression with ME/CFS and PCC [5, 6, 19, 34, 36].

This study received ethical approval from the Griffith University Human Research Ethics Committee (HREC) (Reference Number: 2019/1005) and the Gold Coast University Hospital HREC (Reference Number: HREC/2019/QGC/56469). Informed consent was obtained from all study participants prior to participation. The research design, collection of data and reporting of results have been informed by and adhere to the Australian Government National Health and Medical Research Council National Statement on Ethical Conduct in Human Research 2023 [37], the World Medical Association Declaration of Helsinki [38] and the Strengthening the Reporting of Observational Studies in Epidemiology Statement guidelines [39] (Table S1, Online Resource 1).

### Data collection

The NCNED’s Research Registry Questionnaire was used to collect participants’ sociodemographic characteristics and illness presentation (including symptom presence, severity and frequency) as previously described [22]. This questionnaire also consisted of validated patient-reported outcome measures (PROMs) to quantify participants’ QoL and functional capacity. In the context of the present study, QoL is defined as one’s perceptions of their health, including their physical, mental, emotional and social wellbeing [40, 41]. Functional capacity refers to one’s ability to complete activities relating to daily living and participation in one’s community [42]. QoL and functional capacity were captured in this study via the 36-Item Short-Form Health Survey version 2 (SF-36v2) [41] and the World Health Organization Disability Assessment Schedule version 2.0 (WHODAS 2.0) [42], respectively.

The SF-36v2 [41] consists of eight domains: Physical Functioning, Role Limitations due to Physical Health Problems (Role Physical), Bodily Pain, General Health Perceptions, Vitality, Social Functioning, Role Limitations due to Personal or Emotional Problems (or Role Emotional) and General Mental Health [41]. SF-36v2 [41] domain scores reflect the percentage of QoL, with minimum and maximum scores of 0 and 100, respectively [41].

Seven domains comprise the WHODAS 2.0 [42], including: Cognition, Mobility, Self-Care, Getting Along, Life Activities 1, Life Activities 2 and Participation [42]. The Life Activities 2 domain measures one’s ability to perform work or school activities [42]. Consequently, this domain has been omitted from the present study, as many pwME/CFS and pwPCC experience a reduced capacity to work [9, 16, 34, 43]. WHODAS 2.0 domain scores range from 0 to 100 and correspond to the percentage of disability or difficulty in functioning [42].

### Statistical analyses

Data was analysed using Statistical Package for the Social Sciences (SPSS) version 29 (IBM Corp, Armonk, New York [44]). The statistical methods chosen and the reporting of results have been informed by the Statistical Analyses and Methods in the Published Literature guidelines [45]. For all statistical tests (including post-hoc analyses), α = 0.05 and p-values are accurate to two significant figures except where *p* < 0.001. For all continuous variables, normality and homogeneity of variances were investigated to determine the appropriate parametric or non-parametric test. Normality was confirmed with the Kolmogorov-Smirnov and Shapiro-Wilk tests among pwME/CFS (*n* ≥ 50) and pwPCC (*n* < 50), respectively. Normally distributed variables were assessed for homogeneity of variances using Levene’s test. The number and percentage of participants with missing data are reported for all relevant variables.

### Sociodemographic and illness characteristics

Mann-Whitney *U* tests were used for non-parametric variables and two-tailed independent samples *t*-tests for parametric variables to compare the sociodemographic and illness characteristics of the two study cohorts. Categorical variables were analysed with Chi-square, Fisher’s exact and Fisher-Freeman-Halton tests depending on the distribution and number of outcomes in the dependent variable. Post-hoc pairwise comparisons (either a Chi-square or Fisher’s exact test, depending on the distribution of outcomes) were performed for categorical variables when more than two outcomes returned significance. The p-values resulting from these post-hoc analyses were subsequently adjusted for multiple comparisons using the Benjamini-Hochberg correction [46].

### Multivariate linear regression

For each PROM domain, multivariate linear regression models of symptom presence were generated while controlling for age, sex, body mass index (BMI) and illness duration. Separate regression models were created for the two participant cohorts. The sociodemographic covariates were added into each model using the ‘Enter’ method with entry = 0.05 and removal = 0.10. Using the same entry and removal thresholds, symptoms were subsequently entered into the regression models using the ‘Stepwise’ method. For each PROM domain, the lowest Akaike Information Criterion (AIC) value identified the final model. All independent variables were assessed for multicollinearity and the residuals were examined for outliers and normality, as well as linearity and homoscedasticity with the predictive values. Partial rank correlations of the PROM domains with symptom presence while controlling for age, sex, BMI and illness duration were also performed to determine the robustness of the multivariate linear regression models. The final partial rank correlation models were generated by including the sociodemographic covariates and all symptoms returning significance.

McDonald’s ω internal consistency values were generated for all PROM domains to determine their reliability among the entire study cohort. The threshold for sufficient reliability was ω ≥ 0.7 [47].

### Cluster analysis

An exploratory, agglomerative approach using unsupervised hierarchical cluster analysis was employed to identify clusters among the pwME/CFS and pwPCC collectively based on symptom presence. The hierarchical cluster analysis was performed using Ward’s method and a squared Euclidean distance threshold of 10. Sociodemographic data, illness characteristics and patient-reported outcomes were subsequently compared among the resulting clusters. Categorical variables were analysed using the same methods outlined in the analysis of the sociodemographic and illness characteristics. Non-parametric variables were compared between the clusters with Kruskal-Wallis *H* tests and parametric variables with one-way Analysis of Variance tests. One-way Analysis of Covariance (ANCOVA) tests were employed to compare the parametric PROM domains between the clusters while controlling for age, sex, BMI and illness duration. Partial rank correlations controlling for the sociodemographic covariates were performed for the non-parametric PROM domains and as robustness checks of the parametric domains. The p-values arising from the partial rank correlations were adjusted for multiple comparisons with the Benjamini-Hochberg correction [46].

Internal consistency of the PROM domains was also investigated among each of the clusters using McDonald’s ω and the ω ≥ 0.7 threshold for sufficient reliability [47].

## Results

The present study captured *n* = 60 pwME/CFS and *n* = 30 pwPCC. Two participants included in the previous study [22] – *n* = 1 pwME/CFS and *n* = 1 pwPCC – did not provide complete symptom data and, consequently, could not be included in the cluster or regression analyses of the present study (Fig. 1). The cohort sizes for the regression analyses were *n* = 60 pwME/CFS and *n* = 29 pwPCC, as illness duration data was missing for *n* = 1 pwPCC. Complete symptom data was otherwise available for this participant and included in the cluster analyses.

### Sociodemographic and illness characteristics

Participants’ sociodemographic and illness characteristics are summarised in Table 1. PwME/CFS were significantly younger and had a significantly longer illness duration than pwPCC (*p* = 0.017 and *p* < 0.001, respectively). Most pwME/CFS and pwPCC were female, living in Queensland and had completed tertiary education. ME/CFS case criteria were met by 60.0% (*n* = 18/30) of pwPCC. There was no difference in the distribution of the most stringent ME/CFS criteria met after post-hoc analyses (*p* > 0.05, corrected). PwME/CFS experienced significantly more symptoms compared with pwPCC (*p* < 0.001). Similar results were returned when comparing the total number of symptoms experienced among only the participants fulfilling ME/CFS criteria (*p* = 0.0022).

**Table 1:** Sociodemographic and illness characteristics (all participants)

	PwME/CFS (*n* = 60)	PwPCC (*n* = 30)	*p*
**Age (years**, ***x̄*(*s*)** **[95%CI])** ^a^	41.57 (12.91) [38.23–44.90]	48.13 (10.05) [44.38–51.87]	0.017
Missing	0 (0.0)	0 (0.0)	
**Sex at birth (n (%))** ^b^			0.12
Female	48 (80.0)	19 (63.3)	
Male	12 (20.0)	11 (36.7)	
Missing	0 (0.0)	0 (0.0)	
**BMI (M (Q1–Q3)** **[95%CI])** ^c^	24.20 (21.15–27.40) [22.60–25.70]	26.45 (21.90–28.48) [22.70–27.80]	0.13
Missing	0 (0.0)	0 (0.0)	
**State of residence (n (%))** ^d^			0.33
Australian Capital Territory	0 (0.0)	0 (0.0)	
New South Wales	8 (13.3)	1 (3.3)	
Northern Territory	0 (0.0)	0 (0.0)	
Queensland	44 (73.3)	26 (86.7)	
South Australia	0 (0.0)	0 (0.0)	
Tasmania	0 (0.0)	0 (0.0)	
Victoria	8 (13.3)	3 (10.0)	
Western Australia	0 (0.0)	0 (0.0)	
Missing	0 (0.0)	0 (0.0)	
**Education (n (%))** ^d^			0.60
High school	16 (26.7)	5 (16.7)	
Undergraduate	14 (23.3)	15 (50.0)	
Postgraduate	19 (31.7)	4 (13.3)	
Other	11 (18.3)	6 (20.0)	
Missing	0 (0.0)	0 (0.0)	
**Employment (n (%))** ^b^			<0.001
Not employed	37 (61.7)	2 (6.7)	
** Reason for unemployment (n (%))** ^e^			1.0
Illness	35 (94.6)	2 (100.0)	
Other	2 (5.4)	0 (0.0)	
Missing	0 (0.0)	0 (0.0)	
Employed	23 (38.3)	28 (93.3)	
** Employment status (n (%))** ^d^			<0.001
Casual	8 (34.8)	1 (3.6)	^g^
Part-time	10 (43.5)	5 (17.9)	
Full-time	5 (21.7)	22 (78.6)	^h^
Missing	0 (0.0)	0 (0.0)	
Missing	0 (0.0)	0 (0.0)	
**Illness duration (years**,** M (Q1–Q3)** **[95%CI])** ^c^	10.00 (5.25–18.00) [8.00–14.00]	0.33 (0.25–0.63) [0.25–0.50]	<0.001
Missing	0 (0.0)	1 (3.3)	
**ME/CFS criteria (n (%))** ^d, f^			0.019
None	0 (0.0)	12 (40.0)	
Fukuda	1 (1.7)	3 (10.0)	
CCC [6]	20 (33.3)	8 (26.7)	
ICC [5]	39 (65.0)	7 (23.3)	
**PCC criteria (WHO definition** [19], **n (%))**	0 (0.0)	30 (100.0)	NA
**Total number of symptoms (M (Q1–Q3)** **[95%CI])** ^c^			
All participants	18 (15–20) [17–19]	15 (12–17) [12–16]	<0.001
Participants fulfilling ME/CFS criteria	18 (15–20) [17–19]	15 (12–18) [12–17]	0.0022

*Abbreviations 95%CI* 95% confidence interval; *BMI* Body mass index; *CCC* Canadian Consensus Criteria; *ICC* International Consensus Criteria; *M* Median; *ME/CFS* Myalgic Encephalomyelitis/Chronic Fatigue Syndrome; *NA* Not applicable; *PCC* Post COVID-19 Condition; *PwME/CFS* People with Myalgic Encephalomyelitis/Chronic Fatigue Syndrome; *PwPCC* People with Post COVID-19 Condition; *Q1–Q3* Quartile 1 to quartile 3; *WHO* World Health Organization. ^a^ Analysed with two-tailed independent samples *t*-test. ^b^ Analysed with Chi-square test. ^c^ Analysed with Mann-Whitney *U*-test. ^d^ Analysed with Fisher-Freeman-Halton test. ^e^ Analysed with Fisher’s exact test. ^f^ The distribution of the most stringent ME/CFS criteria met was compared among all participants fulfilling at least the Fukuda case definition (*n* = 60 pwME/CFS and *n* = 18 pwPCC). Participants are categorised by the most stringent ME/CFS criteria fulfilled. ^g^ PwME/CFS > PwPCC ^h^ PwPCC > PwME/CFS

### Symptoms and patient-reported outcomes

The results of all regression models generated, including the unstandardised B coefficients of the included variables and adjusted *R*^2^ goodness-of-fit values, for the SF-36v2 [41] and WHODAS 2.0 [42] subscales are provided in Tables S2 and S3, Online Resource 1 for the pwME/CFS and pwPCC, respectively.

### ME/CFS

Poorer scores in most SF-36v2 [41] domains (including Physical Functioning, Role Physical, Bodily Pain, Vitality and Social Functioning) were observed in the presence of dyspnoea. Additionally, lower Physical Functioning, Role Physical and Social Functioning scores (indicating worsened QoL) were associated with light-headedness or dizziness. Nausea also had negative associations with Physical Functioning, General Health, Social Functioning and Mental Health. Other symptoms returning significant negative associations with SF-36v2 [41] domains included short-term memory loss (Social Functioning), myalgia (Bodily Pain), arthralgia (General Health) and sweating episodes (Role Emotional). Interestingly, muscle weakness returned positive associations with multiple SF-36v2 [41] domains, including Physical Functioning, Role Physical, Vitality and Social Functioning. General Health returned positive relationships with abdominal pain and lymphadenopathy. Headaches were associated with increased Social Functioning scores.

Upon performing robustness checks, myalgia and urinary disturbances gained significance for the Physical Functioning and Vitality models, respectively. Symptoms that lost significance included: muscle weakness (Physical Functioning, Role Physical and Social Functioning), light-headedness (Physical Functioning and Social Functioning), memory loss, headaches and nausea (Social Functioning), arthralgia, abdominal pain and lymphadenopathy (General Health), sweating (Role Emotional) and recurrent feelings of feverishness (Physical Functioning).

Like the regression models for the SF-36v2 [41] domains, dyspnoea was associated with poorer scores in all six WHODAS 2.0 [42] domains included in the present study. Urinary disturbances returned positive associations (indicating heightened disability) with Mobility and Life Activities 1. Worsened Self-Care scores were observed in the presence of light-headedness. Symptoms returning negative associations with WHODAS 2.0 [42] domains included myalgia (Mobility, Self-Care and Life Activities 1), arthralgia (Getting Along and Participation), sleep disturbances (Cognition, Mobility and Life Activities 1), unrefreshed sleep (Mobility and Self-Care), muscle weakness (Self-Care and Participation) and headaches (Cognition).

Following robustness checks, sensitivity to odour or taste and light-headedness gained significance for the Cognition model. Symptoms that lost significance included: sleep disturbances (Cognition and Life Activities 1), unrefreshed sleep (Mobility and Self-Care), muscle weakness (Self-Care and Participation), myalgia and urinary disturbances (Life Activities 1), headaches (Cognition) and arthralgia (Participation).

### PCC

Cold extremities returned the most negative associations with SF-36v2 [41] domains, including Role Physical, Vitality and Social Functioning. Other symptoms that returned poorer scores in more than one SF-36v2 [41] domain included memory loss (Bodily Pain and Mental Health), headaches (Vitality and Social Functioning), dyspnoea (Physical Functioning and Social Functioning) and feverishness (Role Physical and Social Functioning). Additional symptoms returning negative associations included: altered bowel habits and laryngitis (Vitality), abdominal pain (General Health), sleep disturbances (Mental Health), muscle weakness (Physical Functioning) and bloating (Role Emotional). Positive associations with SF-36v2 [41] domains were observed for light-headedness (Bodily Pain and Role Emotional), lymphadenopathy and heart palpitations (General Health) and sensitivity to odour or taste (Bodily Pain).

Upon completing robustness checks, lymphadenopathy gained significance for the Role Physical model but lost significance for General Health. Similarly, altered bowel habits gained significance for Social Functioning but lost significance for Vitality. Other symptoms that lost significance included: headaches (Vitality and Social Functioning), cold extremities (Role Physical and Vitality), sensitivity to odour or taste and light-headedness (Bodily Pain), sleep disturbances (Mental Health), laryngitis (Vitality) and dyspnoea (Social Functioning).

Worsened scores in the Self-Care and Getting Along domains of the WHODAS 2.0 [42] were observed in the presence of abdominal pain. Feverishness was associated with poorer Cognition and Mobility scores. Other symptoms returning positive associations with WHODAS 2.0 [42] domains included memory loss and lymphadenopathy (Cognition), muscle weakness (Self-Care), dyspnoea (Mobility) and sweating (Getting Along). Light-headedness was associated with lower Cognition and Self-Care scores. Lymphadenopathy was a negative predictor of Life Activities 1 and feverishness returned positive associations with both Life Activities 1 and Participation. However, neither the Life Activities 1 nor the Participation regression model were statistically significant (*p* = 0.061 and *p* = 0.15, respectively).

Following robustness checks, feverishness gained significance for the Life Activities 1 model but lost significance for Mobility. Other symptoms that lost significance included: light-headedness (Cognition and Self-Care), abdominal pain and sweating (Getting Along), muscle weakness (Self-Care) and lymphadenopathy (Cognition).

### Reliability statistics

The internal consistency values for each of the PROM domains are summarised in Table S4, Online Resource 1. All WHODAS 2.0 [42] domains returned a McDonald’s ω value greater than 0.7 and therefore had sufficient reliability among pwME/CFS and pwPCC. Most SF-36v2 [41] domains for which internal consistency could be calculated also returned a McDonald’s ω value greater than 0.7 except for Vitality (ω = 0.556).

### Symptom clusters

Four clusters were identified from the hierarchical cluster analysis of symptom presence among the pwME/CFS and pwPCC (Fig S1). The sociodemographic information, illness characteristics and patient-reported outcome data of the four clusters are summarised in Table S5, Online Resource 1. Comparisons of and reliability statistics for the PROM domain scores across the four clusters are provided in Tables S6 and S7, Online Resource 1, respectively.

No significant differences were observed in the sociodemographic characteristics of the four clusters. Across the four clusters, Vitality and Role Physical consistently returned the poorest of the SF-36v2 [41] domain scores and Life Activities 1 and Participation were the greatest affected WHODAS 2.0 [42] domains. Significantly lower scores in the General Health domain of the SF-36v2 [41] were observed for Cluster 2 compared with Cluster 3 (median (M) = 25.00, quartile 1 to quartile 3 (Q1–Q3) = 16.67–31.25, 95% confidence interval (95%CI) = 16.67–25.00 and M = 45.83, Q1–Q3 = 29.17–75.00, 95%CI = 29.17–79.17, respectively; *p* < 0.05, corrected). Cluster 2 also returned significantly higher scores than Cluster 3 in the Cognition domain of the WHODAS 2.0 [42] (M = 55.00, Q1–Q3 = 45.00–60.00, 95%CI = 50.00–55.00 and M = 35.00, Q1–Q3 = 15.00–47.50, 95%CI = 15.00–50.00, respectively; *p* < 0.05, corrected). All PROM subscales for which internal consistency statistics could be generated returned a McDonald’s ω value greater than 0.7 except for the Participation domain of the WHODAS 2.0 [42] (ω = 0.699). The distribution of illness status was not significantly different across the clusters after adjustment for multiple comparisons (*p* > 0.05, corrected). However, the clusters differed significantly in the distribution of the most stringent ME/CFS criteria met (*p* < 0.001, uncorrected), illness duration (*p* = 0.011, uncorrected) and the total number of symptoms (*p* < 0.001, uncorrected).

Complete descriptive statistics of symptom presentation, comparisons between the four clusters and results of post-hoc analyses are summarised in Table S8, Online Resource 1. The distributions of severity and frequency for each symptom among the four clusters are provided in Tables S9 and S10, Online Resource 1, respectively. Across the four clusters, there were no significant differences in the presentation of hallmark ME/CFS symptoms (including post-exertional malaise, impaired concentration and unrefreshed sleep) upon adjustment for multiple comparisons. Muscle weakness was the only symptom to differ in severity across the four clusters (*p* = 0.0057, uncorrected) and no symptoms were significantly different in frequency. However, the prevalence of all thermostatic, cardiovascular and gastrointestinal symptoms, as well as most pain and neurosensory symptoms, differed significantly across the four clusters.

#### Cluster 1

Excluding the hallmark ME/CFS symptoms, the most common symptoms among the Cluster 1 participants included sleep disturbances (*n* = 21/21, 100.0%), altered bowel habits (*n* = 17/21, 81.0%) and myalgia (*n* = 16/21, 76.2%). Symptoms low in prevalence among the Cluster 1 participants included memory loss (*n* = 5/21, 23.8%), lymphadenopathy (*n* = 3/21, 14.3%), palpitations (*n* = 4/21, 19.0%), sweating (*n* = 3/21, 14.3%) and feverishness (*n* = 3/21, 14.3%). Illness presentation was largely comparable between Clusters 1 and 3. Where significant differences between these two clusters existed, most lay in the prevalence of gastrointestinal symptoms.

All neurosensory symptoms (except sensitivity to odour or taste) affected at least half of the Cluster 1 participants. Cluster 1 had a significantly higher prevalence of photophobia (*n* = 15/21, 71.4%) compared with Cluster 3 (*p* < 0.05, corrected). However, the prevalence of muscle weakness was lowest in Cluster 1 (*n* =11/21, 52.4%) and significantly lower when compared with Cluster 2 (*p* < 0.05, corrected). Cluster 1 also returned the lowest prevalence of memory loss, which was significantly less common when compared with Clusters 2 and 4 (both *p* < 0.05, corrected). Cluster 1’s prevalence of arthralgia (*n* = 9/21, 42.9%) and abdominal pain (*n* = 8/21, 38.1%) was significantly less prevalent when compared with Cluster 2 (both *p* < 0.05, corrected).

Altered bowel habits were significantly more common in Cluster 1 compared with Clusters 3 and 4 (both *p* < 0.05, corrected). Bloating was also significantly more prevalent in Cluster 1 than Cluster 3 (*p* < 0.05, corrected). However, nausea was reported by less than half of the Cluster 1 participants and was significantly less prevalent when compared with Cluster 2 (*p* < 0.05, corrected).

Cluster 1’s prevalence of laryngitis (*n* = 11/21, 52.4%), palpitations (*n* = 4/21, 19.0%), light-headedness (*n* = 13/21, 61.9%), sweating (*n* = 3/21, 14.3%) and feverishness (*n* = 3/21, 14.3%) was the lowest of the four clusters – all (except laryngitis) of which were significantly lower compared with Cluster 2 (all *p* < 0.05, corrected). Palpitations and light-headedness were also significantly less common in Cluster 1 than Cluster 3 (both *p* < 0.05, corrected). Lymphadenopathy (*n* = 11/21, 52.4%) was significantly less prevalent in Cluster 1 than Clusters 2 and 4 (both *p* < 0.05, corrected) and was, along with sweating and feverishness, among the least common symptoms experienced by the Cluster 1 participants.

#### Cluster 2

Cluster 2 had the largest symptom burden and returned the highest prevalence of the four clusters for most symptoms. The Cluster 2 participants also experienced significantly more symptoms in total (M=20, Q1–Q3 = 18–22, 95%CI = 19–22 symptoms) when compared with all other clusters (all *p* < 0.05, corrected). Illness duration was longest among the Cluster 2 participants (M = 11.00, Q1–Q3 = 3.50–21.00, 95%CI = 5.00–18.00 years, data missing for *n* = 1 pwPCC from Cluster 2) and significantly longer compared with Clusters 1 and 3 (both *p* <0.05, corrected). Cluster 2 had the highest proportion of participants fulfilling the ICC [5] (*n* = 24/33, 72.7%), which was significantly higher when compared with Cluster 3 (*p* < 0.05, corrected).

Sweating (*n* = 23/33, 69.7%), nausea (*n* = 31/33, 93.9%) and abdominal pain (*n* = 31/33, 93.9%) were significantly higher among Cluster 2 than all other clusters (all *p* < 0.05, corrected). Cluster 2’s prevalence was also significantly higher than that of Clusters 1 and 3 for arthralgia (*n* = 29/33, 87.9%) and lymphadenopathy (*n* = 16/33, 48.5%), Clusters 1 and 4 for muscle weakness (*n* = 32/33, 97.0%), Clusters 3 and 4 for altered bowel habits (*n* =31/33, 93.9%), Cluster 1 alone for memory loss (*n* =26/33, 78.8%), palpitations (*n* = 20/33, 60.6%), light-headedness (*n* = 31/33, 93.9%), sweating, and feverishness (*n* = 18/33, 54.5%) and Cluster 3 alone for myalgia (*n* = 32/33, 97.0%), photophobia (*n* = 28/33, 84.8%), sensitivity to noise or vibration (*n* = 29/33, 87.9%) and bloating (*n* = 25/33, 75.8%) (all *p* < 0.05, corrected). Muscle weakness was more severe for Cluster 2 when compared with Clusters 3 and 4 (both *p* < 0.05, corrected).

#### Cluster 3

Other than the cardinal symptoms of ME/CFS, the most prominent symptoms in Cluster 3 were light-headedness (*n* = 9/9, 100.0%), headaches (*n* = 8/9, 88.9%), muscle weakness (*n* = 6/9, 66.7%), laryngitis (*n* = 6/9, 66.7%) and palpitations (*n* = 6/9, 66.7%). Cluster 3 had the most significant differences with Cluster 2.

Cluster 3’s prevalence of myalgia (*n* = 5/9, 55.6%) was the lowest of the four clusters and significantly lower when compared with Cluster 2 (*p* < 0.05, corrected). Excluding headaches, Cluster 3 had the lowest prevalence of all other pain symptoms, including arthralgia (*n* = 2/9, 22.2%) and abdominal pain (*n* = 1/9, 11.1%) – both of which were significantly less prevalent when compared with Cluster 2 (*p* < 0.05, corrected). All neurosensory symptoms, except muscle weakness, were lowest in prevalence in Cluster 3, including photophobia (*n* = 2/9, 22.2%), sensitivity to noise or vibration (*n* = 2/9, 22.2%) and sensitivity to odour or taste (*n* = 1/9, 11.1%). When compared with Cluster 3, photophobia was significantly more prevalent in Clusters 1, 2 and 4 (all *p* < 0.05, corrected) and sensitivity to noise or vibration was significantly more common in Clusters 2 and 4 (both *p* < 0.05, corrected).

Of the four clusters, Cluster 3 returned the lowest prevalence of all gastrointestinal symptoms. Prevalence was significantly lower among the Cluster 3 participants for nausea (*n* = 1/9, 11.1%) when compared with Clusters 2 and 4, bloating (*n* = 0/9, 0.0%) when compared with Clusters 1, 2 and 4 and altered bowel habits (*n* = 1/9, 11.1%) when compared with Clusters 1 and 2 (all *p* < 0.05, corrected). Among the Cluster 3 participants, prevalence was also significantly lower for lymphadenopathy (*n* = 0/9, 0.0%) when compared with Clusters 2 and 4 and sweating (*n* = 2/9, 22.2%) when compared with Cluster 2 (all *p* < 0.05, corrected).

#### Cluster 4

Cluster 4 was largely comparable with Cluster 2 and had the second-highest median total number of symptoms (M = 16, Q1–Q3 = 14–18, 95%CI = 14–18 symptoms), which was significantly higher when compared with Cluster 3 (*p* < 0.05, corrected). Among the Cluster 4 participants, prevalence was significantly lower than that of Clusters 1 and 2 for altered bowel habits (*n* = 4/27, 14.8%), and Cluster 2 alone for abdominal pain (*n* = 13/27, 48.1%), muscle weakness (*n* = 18/27, 66.7%) and sweating (*n* = 6/27, 22.2%) (all *p* < 0.05, corrected). Altered bowel habits and urinary disturbances were among the least common symptoms in Cluster 4 (both *n* = 4/27, 14.8%). Among the Cluster 4 participants, the prevalence of nausea (*n* = 17/27, 63.0%) and bloating (*n* = 16/27, 59.3%) was significantly higher than that of Cluster 3 but significantly lower than Cluster 2 (all *p* < 0.05, corrected).

## Discussion

The present pilot study documents symptom clusters among pwME/CFS and pwPCC and identifies associations between symptoms and patient-reported outcomes. To the authors’ knowledge, this is the first study to identify symptom clusters in a collective population of pwME/CFS and pwPCC. Importantly, none of the four clusters were specific to ME/CFS or PCC. These novel findings further characterise the illness presentation of PCC to inform developments in the diagnostic criteria of this emergent condition. This study also serves to inform changes to approaches to care and service delivery for pwME/CFS and pwPCC by providing detailed analyses of symptom burden on QoL and functioning. Symptoms were associated with poorer scores in all measures of QoL and functional capacity among both illness cohorts. This reflects the widespread and complex impacts of ME/CFS and PCC on daily living and functioning and, therefore, the need for access to multidisciplinary care and support [1, 3, 7, 8].

Furthermore, this study highlights symptoms that contribute to worsened health outcomes among pwME/CFS and pwPCC, which may signify priority areas for symptom management in clinical practice. Autonomic symptoms appeared the most burdensome and returned poorer patient-reported outcomes across most domains among both pwME/CFS and pwPCC. Interestingly, while negative associations were observed among both illness cohorts, the presence of dyspnoea had a pronounced impact on pwME/CFS. This may be due to case criteria requirements. PwME/CFS must experience a defined set of symptoms to meet diagnostic criteria; however, the presence of respiratory symptoms is not compulsory [5, 6]. Therefore, dyspnoea (in addition to the mandatory, hallmark symptoms of ME/CFS) may further compound existing reductions in QoL and functional capacity.

The contribution of thermostatic intolerance, memory loss, pain, sleep disturbances and muscle weakness to illness burden also varied between pwME/CFS and pwPCC. Thermostatic intolerance was associated with poorer scores in several PROM subscales among pwPCC. However, the only association for this symptom group observed among pwME/CFS was sweating with the Role Emotional domain of the SF-36v2 [41]. Additionally, memory loss was burdensome in both illness cohorts but returned more negative associations with patient-reported outcomes among pwPCC. Furthermore, pain (notably myalgia), muscle weakness and sleep disturbances were associated with increased QoL and functional capacity among pwME/CFS but only returned poor patient-reported outcomes among pwPCC.

These results may be explained by changes in illness presentation over time. Whilst the long-term prognosis of PCC is not yet known, existing ME/CFS literature has noted that the burden of symptoms may evolve as the illness progresses [11, 12]. Among pwME/CFS, the burden of neurocognitive and autonomic dysfunction appears to increase over time and surpass that of flu-like and inflammatory symptoms, which may be more pronounced in earlier stages of illness [11, 12]. This is further supported by the increased total number of symptoms, as well as higher prevalence of gastrointestinal and neurosensory symptoms, observed among clusters with longer illness duration in the present study.

Negative associations between QoL and gastrointestinal symptoms, namely nausea, were also more pronounced among pwME/CFS in this study. Comorbid gastrointestinal disorders appear common in ME/CFS, with up to 60% of pwME/CFS experiencing IBS [48, 49]. New-onset IBS has also been disproportionately observed among COVID-19 survivors [50]. The pathophysiological mechanisms underpinning gastrointestinal symptoms in ME/CFS and PCC remain unclear. Additionally, the burden of gastrointestinal symptoms among pwPCC varies in the existing literature and has been described as both comparable to and less than that experienced by pwME/CFS [22, 51, 52]. Nevertheless, the results of the present study reiterate the importance of identifying and managing potential comorbid gastrointestinal disorders and related symptoms (as outlined in clinical guidelines for ME/CFS [4, 9, 53, 54]) to reduce further implications on QoL.

It is also worth noting that, as some symptoms that returned positive associations with patient-reported outcomes were highly prevalent, the sample sizes of participants not experiencing these symptoms were small. Consequently, the relative increases in QoL and functional capacity among these participants may not be truly representative. Importantly, the associations between symptoms and patient-reported outcomes observed in the present study are relative to the presence of other symptoms and positive associations should not be interpreted as minimising the burden of these symptoms on the lives of pwME/CFS and pwPCC.

Despite some differences in the regression analyses, pwME/CFS could not be distinguished from pwPCC through hierarchical cluster analysis of symptom presence. There were no significant differences in the prevalence, severity or frequency of hallmark ME/CFS symptoms across the four clusters. Differences in patient-reported outcomes across the four clusters were marginal. The Cognition domain of the WHODAS 2.0 [42] was significantly more impaired among the Cluster 2 than Cluster 3 participants. Friedberg et al. [55] observed a worsening of cognitive symptoms among pwME/CFS over time. However, Cluster 2 (while characterised by a significantly longer illness duration) did not differ from Cluster 3 in the prevalence, severity or frequency of cognitive disturbances. The disparities in the scores for the General Health domain of the SF-36v2 [41] among the Cluster 2 and Cluster 3 participants likely align with the differences in illness duration [12, 56], as the General Health subscale queries self-perceptions of health in comparison to others, as well as self-perceptions of future health [41].

Clusters 2 and 3 had the lowest comparability of all the clusters. These two clusters were the only cluster pair to differ significantly in measures of QoL and functional capacity, as well as in the distribution of fulfilling the ICC [5] case definition for ME/CFS. The likelihood of spontaneously recovering from ME/CFS is highest within the first two years following illness onset [11, 12]. This could explain the lower prevalence of many symptoms in Cluster 3, which returned the shortest median illness duration of 1.58 years. Hence, these cluster analysis findings may suggest that the illness burden of ME/CFS and PCC accumulates over time. Similarly, as the four symptom clusters appeared to be differentiated largely by the presence of gastrointestinal and neurosensory disturbances, an increased burden of these symptoms may potentially indicate a later stage of illness.

This postulation is supported by the regression results of the present study, as well as the increased burden of autonomic and neurosensory symptoms as ME/CFS progresses [11, 12]. Following cluster analyses of pwME/CFS fulfilling the Fukuda criteria [57], Słomko et al. [58] also documented poorer QoL and worsened fatigue in a cluster defined by autonomic dysfunction. Participants in this cluster returned a higher burden of autonomic symptoms, poorer scores in autonomic function tests, greater fatigue impact and severity, higher prevalence of post-exertional malaise and the lowest QoL of the four identified clusters [58].

Publications investigating clusters among pwPCC have similarly identified subgroups delineated by the prevalence of flu-like symptoms and pain when compared with neurocognitive and gastrointestinal symptoms [31, 33, 59–61]. However, further longitudinal research is necessary to determine whether the contributors to PCC burden shift to resemble those of ME/CFS over time. Cluster analyses among COVID-19 survivors have suggested that a greater number of symptoms and increased symptom severity during acute SARS-CoV-2 infection may be associated with an increased likelihood of PCC development, as well as a heightened PCC symptom burden [33, 60, 61]. However, the indicators of recovery from or persistence of PCC have not been well-described. The present paper therefore provides novel insight into the potential illness trajectory of PCC. Future longitudinal research is warranted to determine the role of post-acute symptom presentation in predicting long-term PCC prognosis.

## Strengths and limitations

There are limitations to this study, including the small sample size and cross-sectional nature. To confirm the role of illness duration in the differences observed in the present study, pwPCC should be matched with pwME/CFS with comparable illness durations in future studies to further evaluate PCC as an equivalent model to the early stages of ME/CFS. Additionally, the contributions of each symptom to patient-reported outcomes reported in this study are relative to other symptoms. Hence, the regression analyses reported herein do not convey the crude impact of ME/CFS and PCC symptoms (particularly hallmark ME/CFS symptoms, which were ubiquitous among the study population) on QoL and functional capacity. Finally, the results presented in this study may not accurately represent pwME/CFS and pwPCC who belong to marginalised populations.

The use of validated PROMs to mitigate observer bias was a strength of the present investigation. This study also benefited from having multiple methods of survey administration. Participants were able to complete the questionnaire online, as an offline digital or paper copy, or over the phone to allow for flexibility in participation. The questionnaire was also suitable to be completed with assistance from a family member, friend or support worker and could be paused and recommenced at any time to enable participants experiencing severe illness to participate in the research.

Importantly, this study provides detailed analyses of the illness presentation of ME/CFS and PCC to inform care pathways for people who live with these conditions. The impairments in all patient-reported outcomes observed in this study necessitate access to multidisciplinary care services for pwME/CFS and pwPCC. Additionally, as autonomic symptoms (such as respiratory and gastrointestinal issues) were noteworthy contributors in the regression and cluster analyses, the present study highlights the potential importance of prioritising these symptoms in the management of ME/CFS and PCC to prevent further deteriorations in QoL and functioning. This study also documents novel data among an Australian cohort of pwME/CFS and pwPCC to inform national health policies that determine access to care and support services. Finally, the findings of this study also serve to inform PCC case definitions. The noteworthy similarities between these conditions observed in this study reiterate the importance of identifying pwPCC experiencing ME/CFS-like illness to deliver early interventions and optimise health outcomes.

## Conclusion

Autonomic symptoms appear to contribute to a worsened illness burden among both pwME/CFS and pwPCC. However, associations of thermostatic intolerance, memory loss, pain, sleep disturbances and muscle weakness with QoL and functional capacity varied between the two illness cohorts. While four symptom clusters were identified, none were unique to ME/CFS or PCC. This further implicates ME/CFS as a potential post-infectious sequela of acute COVID-19 illness and reiterates the possible benefit of shared management approaches. Longitudinal research is warranted to confirm the relationship between cluster status and illness progression. Meanwhile, the numerous negative associations between symptoms and patient-reported outcomes in the present study emphasises the importance of multidisciplinary and person-centred care and support for all pwME/CFS and pwPCC.

## Electronic supplementary material

Below is the link to the electronic supplementary material.

Supplementary Material 1

Supplementary Material 2

## Data Availability

The datasets generated and analysed that support the findings of the current study are not publicly available due to confidentiality agreements. However, these datasets are available from the corresponding author upon reasonable request.

## Abbreviations

95%CI: 95% confidence interval
AIC: Akaike Information Criterion
ANCOVA: Analysis of Covariance
BMI: Body mass index
C: Consensus
CCC: Canadian Consensus Criteria
HREC: Human research ethics committee
IBS: Irritable Bowel Syndrome
ICC: International Consensus Criteria
M: Median
ME/CFS: Myalgic Encephalomyelitis/Chronic Fatigue Syndrome
NA: Not applicable
NCNED: National Centre for Neuroimmunology and Emerging Diseases
PCC: Post COVID-19 Condition
PROM: Patient-reported outcome measure
PwME/CFS: People/person with Myalgic Encephalomyelitis/Chronic Fatigue Syndrome
PwPCC: People/person with Post COVID-19 Condition
Q1–Q3: Quartile 1 to quartile 3
QoL: Quality of life
SARS-CoV-2: Sudden Acute Respiratory Syndrome Coronavirus-2
SF-36v2: 36-Item Short-Form Health Survey (version 2)
SPSS: Statistical Package for the Social Sciences
WHO: World Health Organization
WHODAS 2.0: World Health Organization Disability Assessment Schedule (version 2.0)
