# Supplementary figures and images for: A pilot cross-sectional investigation of symptom clusters and associations with patient-reported outcomes in Myalgic Encephalomyelitis/Chronic Fatigue Syndrome and Post COVID-19 Condition
Source: Qual Life Res. 2024 Oct 3;33(12):3229–43. doi: 10.1007/s11136-024-03794-x (PMC11599292; doi:10.1007/s11136-024-03794-x)

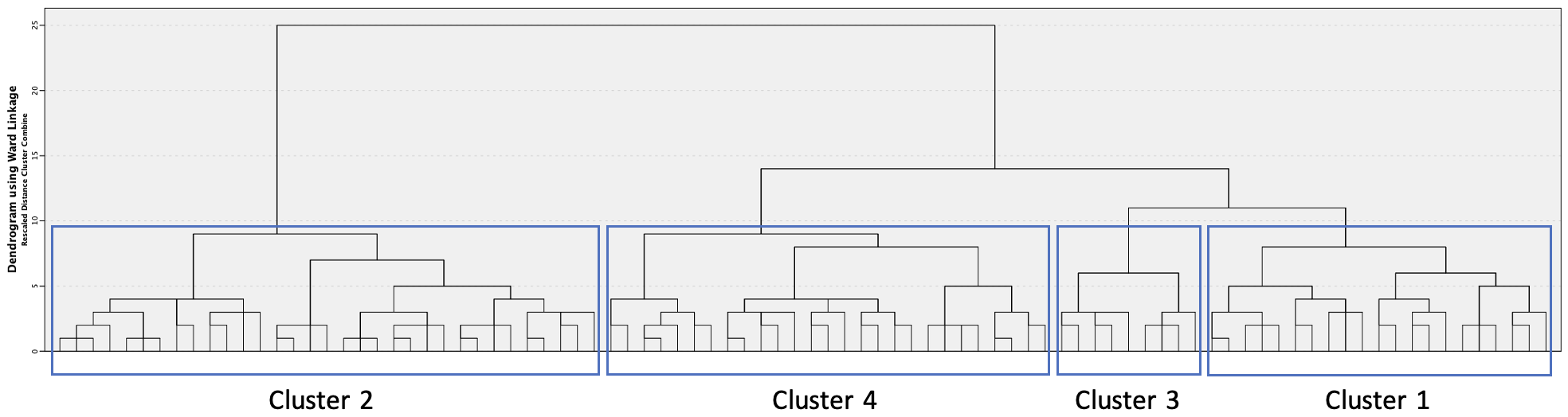

Supplement: Supplementary file 2 — Supplementary Material 2 [file 11136_2024_3794_MOESM2_ESM.tif]
